# Diagnosis of Pulmonary Injury During Laparoscopic Cholecystectomy: A Case Report

**DOI:** 10.7759/cureus.88306

**Published:** 2025-07-19

**Authors:** Lucas Ricchetti, Filipe Golart, Marcio Losso, Luiz F Soares, Danilo Hantschick Fernandes Monteiro

**Affiliations:** 1 Department of Cardiac Anesthesiology, Hospital SOS Cardio de Santa Catarina, Florianópolis, BRA; 2 Principles and Practice of Clinical Research (PPCR) Program, Harvard T. H. Chan School of Public Health, Florianopolis, BRA

**Keywords:** anesthesiologist role, critical event recognition, intraoperative assessment, intraoperative ultrasound, laparoscopic cholecystectomy, traumatic diaphragmatic injury

## Abstract

Laparoscopic cholecystectomy (LC) is widely regarded as the standard treatment for acute cholecystitis, particularly in emergency settings. Although it is a minimally invasive procedure with a relatively high safety profile, intraoperative complications can occur, some of which are rare but potentially life-threatening. We report the case of an 86-year-old male patient who underwent emergency LC for acute cholecystitis, during which an iatrogenic injury to the right hemidiaphragm with ipsilateral lung involvement was identified. This complication resulted in a pneumothorax, necessitating immediate chest drainage. In this context, the anesthesiologist plays a critical role in the early recognition of pneumothorax through bedside lung ultrasonography, enabling prompt diagnosis and intervention. This case highlights the importance of early detection and a multidisciplinary approach in managing uncommon intraoperative complications.

## Introduction

Laparoscopic cholecystectomy (LC), a minimally invasive surgical technique, has become the gold standard for treating symptomatic gallbladder disease, including acute cholecystitis. First introduced in the late 1980s, LC rapidly replaced open cholecystectomy due to its numerous advantages such as reduced postoperative pain, shorter hospital stays, faster recovery, and decreased risk of wound complications [1]. Despite its favorable safety profile, LC is not without risk. The most common complications include bile duct injury, hemorrhage, and infection. However, thoracic complications, such as pneumothorax, although rare, are clinically significant and may be underrecognized. These complications can occur due to diaphragmatic injury during the insertion of laparoscopic trocars or from dissection near the hepatic dome, where the diaphragm is closely related to the gallbladder and can lead to gas migration into the thoracic cavity. Furthermore, elevated airway pressures resulting from capnoperitoneum may reduce cardiac output and contribute to barotrauma such as interstitial emphysema and pneumothorax [2]. Early identification of such complications is essential to avoid further deterioration. Anesthesiologists play a critical role in recognizing abnormal ventilatory parameters such as increased airway pressures or end-tidal carbon dioxide (ETCO₂), reduced lung compliance, or asymmetrical breath sounds. Recently, the use of bedside lung ultrasonography has gained traction as a fast, sensitive, and specific tool for the diagnosis of pneumothorax in the intraoperative setting, especially when radiographic modalities are not immediately available [3,4]. In this case report, we describe a rare but serious complication: iatrogenic diaphragmatic and pulmonary injury during LC, resulting in intraoperative tension pneumothorax. This case emphasizes the importance of intraoperative vigilance, effective communication between surgical and anesthetic teams, and the incorporation of point-of-care ultrasonography (POCUS) in the diagnosis and management of thoracic complications during laparoscopic procedures.

## Case presentation

An 86-year-old male weighing 60 kg, with a body mass index (BMI) of 29.0 kg/m², was admitted to the operating room for emergency LC due to acute cholecystitis. The patient's medical history included systemic arterial hypertension, for which he was taking losartan and hydrochlorothiazide. He had a functional capacity of less than 4 metabolic equivalents (METs), was classified as American Society of Anesthesiologists (ASA) physical status III, and had a Frailty Index of 2, consistent with a pre-frail status. There was no history of structural lung disease.

Initial monitoring showed noninvasive blood pressure of 212/79 mmHg, peripheral oxygen saturation (SpO₂) of 92% on room air, which was attributed to reduced functional capacity (<4 METs) and probable atelectasis, a common finding in elderly patients. The respiratory rate (RR) was 14 bpm, and the heart rate (HR) was 86 bpm in sinus rhythm. The preoperative chest X-ray was normal, with clear lung fields and no evidence of pleural effusion, consolidation, or structural abnormalities.

After adequate pre-oxygenation, anesthesia was induced with a continuous infusion of remifentanil at 0.1 mcg/kg/min, 20 mg lidocaine, 110 mg propofol, and 30 mg rocuronium (neuromuscular blockade monitored via train of four (TOF)). Orotracheal intubation was uneventful and confirmed by capnography and lung auscultation. Anesthesia was maintained with 1.5% sevoflurane using a mixture of oxygen and air and remifentanil at 0.2 mcg/kg/min. The mechanical ventilation parameters were volume-controlled ventilation at 450 ml, RR of 14 bpm, positive end-expiratory pressure (PEEP) of 5 cmH₂O, inspiratory pause of 20% (peak pressure 20 cmH₂O, plateau pressure 13 cmH₂O, driving pressure 8 cmH₂O), fraction of inspired oxygen (FiO₂) of 50%, and pressure limit of 35 cmH₂O.

The surgical procedure began with the patient in the dorsal decubitus position, the creation of pneumoperitoneum using CO₂ at 15 mmHg, and standard positioning for LC (reverse Trendelenburg and left tilt). Initial intraoperative parameters were blood pressure (BP) of 130/65 mmHg, HR of 80 bpm, RR of 14 bpm, SpO₂ of 100%, ETCO₂ of 36 mmHg, and peak pressure of 26 cmH₂O.

Approximately 30 minutes after the procedure began, a sudden and progressive increase in both peak airway pressure and ETCO₂ was observed. The ventilator reached its preset pressure limit, with a reduction in delivered tidal volume and an ETCO₂ elevation to 50 mmHg. On auscultation, there was a notable reduction in breath sounds on the right and the emergence of bilateral wheezing, which had not been present earlier.

Given the clinical picture, endobronchial intubation due to patient repositioning was initially suspected. During evaluation, direct laryngoscopy confirmed the cuff of the orotracheal tube positioned just below the vocal cords. Simultaneously, the anesthesia machine signaled a "circuit leak", yet no disconnections or external leaks were detected upon inspection, and the patient experienced a drop in SpO₂ to 86%. Ventilator graphics showed a rightward deviation in the pressure-volume loop with decreased compliance, consistent with increased airway pressures and possible intrathoracic pathology. Differential diagnoses considered included bronchospasm, mucus plugging, and intrathoracic pathologies such as pneumothorax.

**Figure 1:**
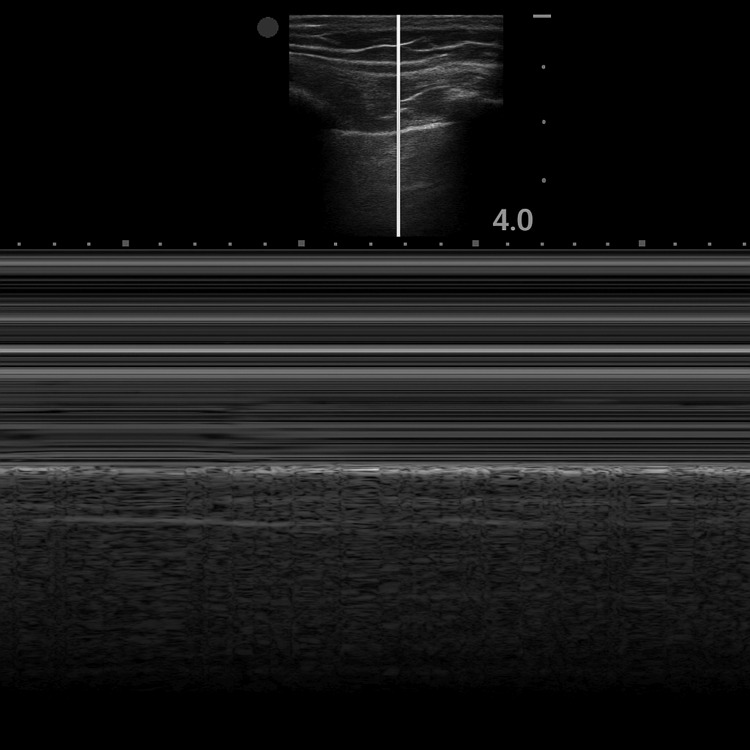
M-mode ultrasound demonstrating the seashore sign in normal lung sliding M-mode lung ultrasound image demonstrating the seashore sign, characterized by parallel horizontal lines in the upper portion of the image (representing the static chest wall) and a granular pattern in the lower portion (representing lung movement). This finding indicates preserved pleural sliding, effectively ruling out pneumothorax at the scanned site.

**Figure 2:**
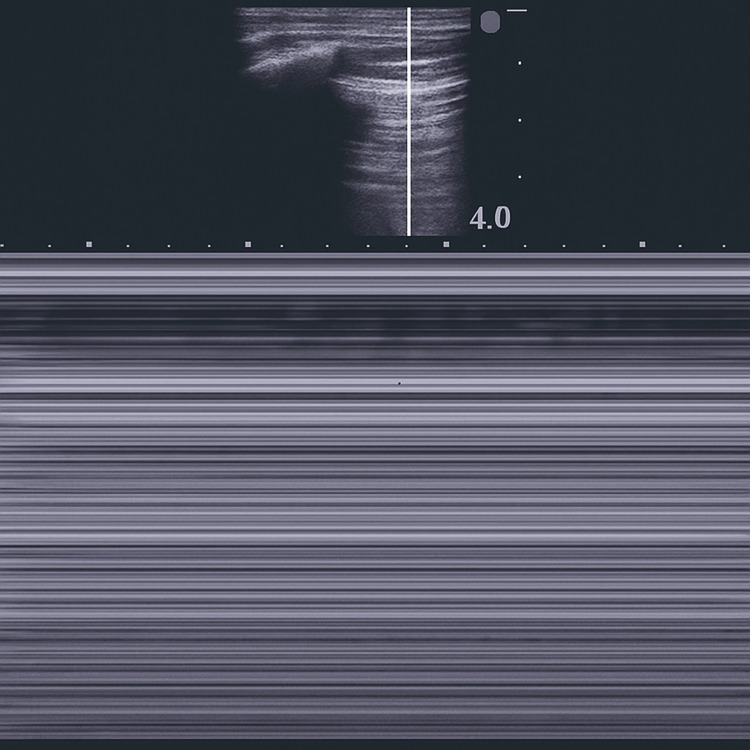
M-mode ultrasound demonstrating the barcode sign in pneumothorax M-mode lung ultrasound image showing the barcode sign (also known as the stratosphere sign), characterized by a pattern of horizontal lines throughout the image, without the granular appearance seen in normal lung sliding. This pattern reflects absent pleural sliding, which is highly suggestive of pneumothorax at the examined site.

After the rise in airway pressure and ETCO₂, as the first treatment measure, 100% oxygen was administered and surgical pneumoperitoneum pressure was reduced. Following the onset of clinical signs, an intraoperative ultrasonography was subsequently employed to assist in establishing the diagnosis. The left lung demonstrated lung sliding and a seashore sign (Figure 1), while the right lung exhibited absent lung sliding and a barcode sign (Figure 2). These findings strongly suggested the presence of a pneumothorax resulting from an inadvertent diaphragmatic injury during laparoscopy, most likely caused by the insertion or manipulation of the trocar. Upon careful inspection, the surgeon identified a 1-cm linear defect in the right hemidiaphragm, which was immediately sutured with absorbable stitches. This led to a temporary improvement in ventilation, resolution of wheezing, and the airway pressure decreased to 20 cmH2O. A lung-protective ventilation strategy was implemented to minimize further barotrauma. However, shortly after, ventilatory parameters worsened again, with new increases in peak pressure and ETCO₂, and absence of breath sounds throughout the right hemithorax. A pulmonary parenchymal injury and right pneumothorax were suspected. A chest tube was inserted in the fifth intercostal space at the mid-axillary line and connected to a water seal drainage system. Movement of the water column was observed, confirming adequate pleural drainage and tube patency. Ventilation parameters subsequently normalized. The patient remained hemodynamically stable and did not experience desaturation at any point.

The surgery proceeded for an additional 2.5 hours without further complications. The patient was extubated at the end of the procedure and transferred to the post-anesthesia care unit (semi-intensive) with nasal oxygen at 2 L/min, stable vital signs, eupneic, and asymptomatic. A postoperative chest radiograph was performed to assess and confirm proper positioning and function of the chest drainage. The clinical course over the following four days was stable, with progressive improvement in SpO₂. The chest tube was removed prior to hospital discharge, and complete re-expansion of the affected lung was achieved without the need for prolonged hospitalization.

## Discussion

Although rare, pneumothorax during laparoscopic surgery is a potentially life-threatening complication that requires early recognition by the anesthesiologist. Incidence may reach up to 1.9%, with risk factors including advanced age, ETCO₂ ≥50 mmHg, prolonged surgical time (>200 minutes), and inexperienced surgeons. A history of lung disease, barotrauma, and laparoscopic surgery increases the risk of developing intraoperative pneumothorax. The diagnosis during surgery could be difficult because the signs are often nonspecific [5].

Pneumothorax may result from spontaneous rupture of emphysematous bullae during mechanical ventilation or CO₂ leakage from the pneumoperitoneum into the pleural space via congenital diaphragmatic defects or weak points (e.g., around the aorta, vena cava, or esophagus), leading to pneumomediastinum and subsequent pneumothorax. Laparoscopic surgery itself is considered a risk factor for developing pneumothorax [6]. Pneumothorax during laparoscopic cholecystectomy may result from direct trauma to the diaphragm or lung, either during dissection of the gallbladder from the hepatic bed or trocar insertion [6,7]. In the present case, the mechanism was most likely an inadvertent diaphragmatic injury caused by trocar placement.

While chest radiography is useful for confirming the diagnosis in both outpatient and inpatient settings, and intraoperative fluoroscopy may provide radiologic confirmation, these methods depend on dedicated equipment and cannot be performed promptly in most general operating rooms [8].

Thoracic ultrasound is readily available and is regarded as nearly equivalent to computed tomography in terms of diagnostic accuracy [3,9]. M-mode ultrasound can display the characteristic “seashore sign” due to the presence of pleural sliding. This phenomenon occurs when the visceral and parietal pleura are in direct contact, producing a side-to-side shimmering motion along the pleural line. In healthy lung parenchyma with adequate ventilation, this results in a granular, seashore-like pattern on M-mode imaging [10]. When gas accumulates between the pleural layers, as in pneumothorax, this sliding motion is abolished. In such cases, lung sliding is absent, and the “lung point” - the transition zone between sliding and non-sliding pleura - may become visible [3]. When the lung is compressed and motionless, the pleural interface instead displays the “barcode sign” or “stratosphere sign” [11]. This finding allows for the rapid and accurate diagnosis of pneumothorax.

Incorporating pulmonary ultrasound into anesthetic practice enhances diagnostic agility, supports timely clinical decision-making, and may significantly reduce morbidity associated with delayed recognition of intrathoracic complications. The anesthesiologist is uniquely positioned to perform this assessment, given their continuous presence at the patient’s side and familiarity with cardiorespiratory physiology. During the laparoscopic procedure, the surgical team identified a small diaphragmatic laceration, which was promptly repaired with sutures, confirming the suspected etiology of the pneumothorax. In this case, the pneumothorax was rapidly diagnosed by the anesthesia team using a bedside lung ultrasound protocol, which allowed for early recognition and timely management before hemodynamic compromise occurred [4]. A lung-protective ventilation strategy was subsequently implemented, including reduced tidal volumes and limited airway pressures, to minimize further barotrauma and support gas exchange.

In this case, progressive ventilatory changes, circuit leak alarms, and absence of breath sounds strongly suggested an underlying pulmonary injury and the possibility of pneumothorax. After chest drainage, continuous bubbling in the water-seal chamber indicated a persistent air leak, likely from the lung.

Pneumothorax in LC exacerbates the physiological effects of pneumoperitoneum (atelectasis, reduced functional residual capacity, increased airway pressures, hypercarbia), especially dangerous in elderly patients with limited cardiopulmonary reserve. Diagnosis is typically made through identification of atypical ventilatory changes (e.g., rising airway pressures and ETCO₂), comparative auscultation, and can be supported by point-of-care lung ultrasound, which offers high sensitivity and specificity for pneumothorax detection.

The decision to proceed with surgery should only be made after the underlying issue has been identified and addressed, taking into account both the patient's hemodynamic stability and the urgency of the procedure [12]. Effective communication between anesthesia and surgical teams is crucial for prompt management. This case underscores the importance of anesthesiologists being trained in POCUS for real-time diagnosis of intraoperative thoracic complications.

## Conclusions

This case highlights the paramount importance of timely diagnosis and immediate intervention in cases of intraoperative pneumothorax associated with laparoscopic surgery. Although rare, iatrogenic diaphragmatic and pulmonary injuries can occur even in routine surgeries such as laparoscopic cholecystectomy. The use of point-of-care lung ultrasound by the anesthesia team played a pivotal role in the timely diagnosis, enabling immediate surgical correction and chest drainage. This facilitated hemodynamic stability, avoided further complications, and ensured a favorable outcome. Incorporating bedside lung ultrasonography into standard anesthetic practice may significantly enhance patient safety by allowing for rapid identification and treatment of potentially life-threatening thoracic complications.
